# Successful Kidney Transplantation in a Recipient Coinfected with Hepatitis C Genotype 2 and HIV from a Donor Infected with Hepatitis C Genotype 1 in the Direct-Acting Antiviral Era

**DOI:** 10.1155/2020/7679147

**Published:** 2020-01-29

**Authors:** Dimitrios Farmakiotis, Zoe Weiss, Amy L. Brotherton, Paul Morrissey, Reginald Gohh, Kendra Vieira, Lynn E. Taylor, Joseph M. Garland

**Affiliations:** ^1^Division of Infectious Diseases, Department of Internal Medicine, Brown Alpert Medical School, Providence, RI, USA; ^2^Department of Internal Medicine, Brown Alpert Medical School, Providence, RI, USA; ^3^Division of Infectious Diseases, Brigham and Women's Hospital and Massachusetts General Hospital, Harvard Medical School, Boston, MA, USA; ^4^Department of Pharmacy, The Miriam Hospital, Providence, RI, USA; ^5^Department of Surgery (Transplantation), Brown Alpert Medical School, Providence, RI, USA; ^6^Division of Nephrology (Transplantation), Department of Internal Medicine, Brown Alpert Medical School, Providence, RI, USA; ^7^CODAC Behavioral Health, Providence, RI, USA

## Abstract

Despite significant advances in transplantation of HIV-infected individuals, little is known about HIV coinfected patients with hepatitis C virus (HCV) genotypes other than genotype 1, especially when receiving HCV-infected organs with a different genotype. We describe the first case of kidney transplantation in a man coinfected with hepatitis C and HIV in our state. To our knowledge, this is also the first report of an HIV/HCV/HBV tri-infected patient with non-1 (2a) HCV genotype who received an HCV-infected kidney graft with the discordant genotype (1a), to which he converted after transplant. Our case study highlights the following: (1) transplant centers need to monitor wait times for an HCV-infected organ and regularly assess the risk of delaying HCV antiviral treatment for HCV-infected transplant candidates in anticipation of the transplant from an HCV-infected donor; (2) closer monitoring of tacrolimus levels during the early phases of anti-HCV protease inhibitor introduction and discontinuation may be indicated; (3) donor genotype transmission can occur; (4) HIV/HCV coinfected transplant candidates require a holistic approach with emphasis on the cardiovascular risk profile and low threshold for cardiac catheterization as part of their pretransplant evaluation.

## 1. Background

Contemporary cohorts of HIV-infected transplant recipients have demonstrated excellent patient and graft survival [1–7]. Similarly, for patients with hepatitis C virus (HCV) infection, kidney transplantation offers a survival benefit and can be more cost-effective than remaining on the waitlist [8–10]. However, compared to their HCV-uninfected counterparts, these patients experience higher rates of post-transplant glomerulonephritis, malignancy, and progression of liver disease [11, 12].

Historically, HCV eradication was a challenge in transplanted patients, as interferon-containing regimens were relatively contraindicated due to increased risk of rejection [13]. The introduction of direct-acting antivirals (DAA) allows for safe treatment of HCV-infected transplant recipients, and therefore, transplantation of HCV-infected organs to both HCV-infected and uninfected patients [9, 14–19]. The optimal timing for treatment of HCV-infected transplant candidates (pre- vs. post-transplant with HCV-infected or HCV-uninfected organ donation, respectively) remains controversial, as the risk of treatment delay must be balanced by the benefit of shorter wait time for HCV-infected organs [7, 10, 13]. DAA prophylaxis for uninfected recipients of HCV-infected organs, including shorter courses [17], has also been proposed as an effective way to expand the organ pool [16–18].

Given similar routes of transmission, coinfection with hepatitis B virus (HBV) or HCV is common among HIV-infected patients with end-stage renal disease (ESRD) [5]. Higher overall morbidity, mortality, and accelerated hepatic decompensation have been observed in this population, compared to HIV monoinfected patients [20, 21], likely due to the immune modulating effects of HCV [22], including activation of CD4+ and CD8+ cells [23] and cytokine production [24]. In the pre-DAA era, transplantation in patients coinfected with HIV and HCV was associated with reduced graft survival and higher rates of serious infection [25]. However, recent small case series showed improved outcomes, including fewer infectious complications, in coinfected patients treated with DAA after transplant [3, 4, 6].

Despite significant advances in transplantation of HIV/HCV coinfected individuals, little is known about coinfected patients with HCV genotypes other than genotype 1, especially when receiving HCV-positive organs with a different genotype. Herein, we describe the first case of kidney transplantation in a man coinfected with hepatitis C and HIV in the State of Rhode Island. To our knowledge, this is also the first reported case of discordant HCV genotype transplantation in a patient coinfected with HIV, in the DAA era.

## 2. Case Report

A 64-year-old man with ESRD from diabetic nephropathy on hemodialysis for one year presented for transplant evaluation. He had a remote history of polysubstance addiction including heroin injection with methadone detoxification and then complete nonuse of illicit drugs for decades; he did not want opioid agonist therapy and was active in Narcotics Anonymous for years. He had a 30 pack-year smoking history, but had quit tobacco at the same time as illicit drugs.

He had a history of well-controlled HIV diagnosed in 1987, with an undetectable viral load and CD4-infected cell count >500 cells/mm^3^ for many years. He had been on numerous prior antiretroviral regimens to treat his HIV and harbored multiclass resistance to agents in the nonnucleoside reverse transcriptase inhibitor (NRTI: M184V and T215N/S/Y) and protease inhibitor (PI: L33I, M46I, I54V, I62V, and V82A/I/T) classes. He ultimately achieved long-term virologic suppression with etravirine, darunavir, ritonavir, and raltegravir (the TRIO [26, 27] regimen). In advance of transplant, his resistance history was reviewed and an HIV-1 proviral DNA genotype archive was sent, which did not demonstrate any resistance to the integrase inhibitor or the NNRTI class. He was switched to rilpivirine and dolutegravir [28] to reduce the risk of drug-drug interactions (DDI) (ritonavir significantly increases calcineurin inhibitor (CNI) levels) and to avoid complex dose adjustment with fluctuating renal function post-transplant.

The patient was also infected with HBV. Tenofovir disoproxil fumarate had been discontinued several years before due to concern for contribution to his progressive renal disease, and he was maintained on lifelong entecavir treatment. He also had chronic hepatitis C virus (HCV), genotype 2b, and declined interferon therapy for years. By the time DAAs became available, there was no DAA for genotype 2 that could be given with his degree of advanced renal disease. When he developed advanced fibrosis (F3), it was recommended that consideration of renal transplant be accelerated given advancing liver disease. He had a history of coronary artery disease (CAD) and inferior myocardial infarction eight years before, with a stent placed to the right coronary artery (RCA). He was taking aspirin 325 mg daily and atorvastatin 40 mg daily. As part of his pretransplant evaluation, a nuclear myocardial perfusion test revealed a large-sized inferior and inferolateral fixed defect with preserved ejection fraction. The patient had very good functional status and did not report any symptoms, so further preoperative cardiac workup was not pursued.

**Figure 1:**
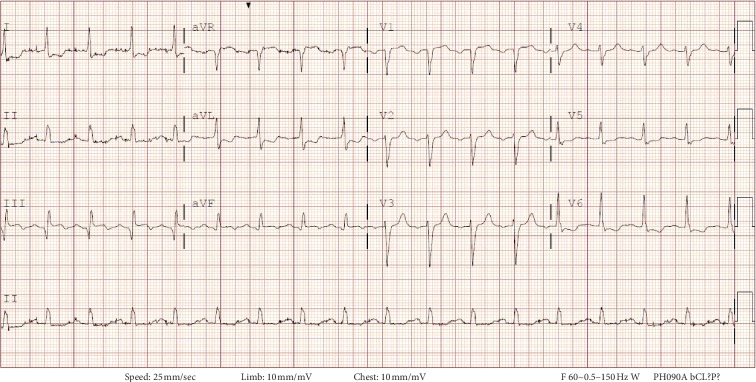
Electrocardiogram during an episode of chest pain on POD 1, showing ST-segment inferior wall elevation and lateral wall depression, concerning for acute injury.

**Figure 2:**
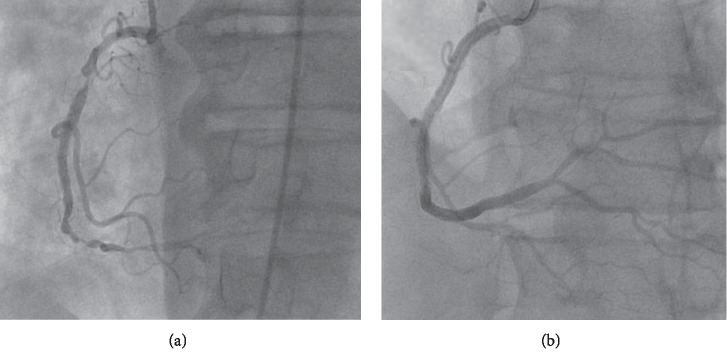
Coronary angiography showing near-complete occlusion of the RCA stent (a) on POD 3 and restored patency after placement of two DES on POD 10 (b).

Three months after listing, he underwent deceased donor kidney transplant from an HCV-positive donor (by antibody and nucleic acid-testing (NAT)-genotype 1a) with basiliximab induction. Maintenance immunosuppression included mycophenolate sodium, tacrolimus, and prednisone. He was also started on valganciclovir and atovaquone prophylaxis due to sulfonamide allergy. On postoperative day (POD) 1, he developed substernal anginal chest pain. An electrocardiogram (EKG) showed ST depressions in the anterolateral leads and transient ST elevation in lead III (Figure 1). His serum troponin peaked at 31 ng/ml (normal <0.06 ng/ml). He was treated with aspirin, clopidogrel, and nitroglycerin drip, without anticoagulation, for fear of hemorrhage due to extensive retroperitoneal blunt dissection during transplant. He underwent cardiac catherization on POD3, which revealed 3-vessel disease, with near-complete occlusion of the RCA stent (Figure 2(a)). On POD 10, he had successful placement of two drug-eluting stents in the RCA (Figure 2(b)). The patient experienced delayed graft function, requiring hemodialysis but was eventually discharged off dialysis on POD 15. Of note, on POD 9 his HCV genotype was 2b, but subsequent genotype before initiation of treatment (POD 81) was only 1a (donor-derived). On POD 100, the patient was admitted for febrile neutropenia, requiring colony-stimulating growth factor support and discontinuation of both valganciclovir and mycophenolate.

**Figure 3:**
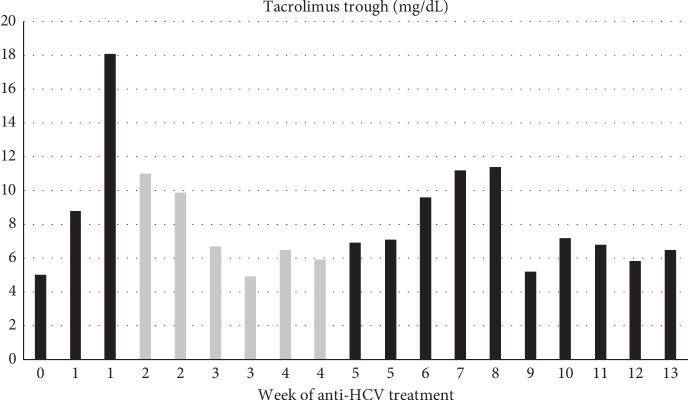
Tacrolimus trough level variations during treatment for hepatitis C with glecaprevir/pibrentasvir and tacrolimus dose: 2 mg bid (black) and 1 mg bid (gray).

On POD 141, he was started on therapy for HCV, with pangenotypic glecaprevir/pibrentasvir (GLE/PIB) for 12 weeks, targeting both donor and recipient genotypes. Concomitant use of GLE/PIB and atorvastatin is contraindicated; thus, he was switched to pravastatin during treatment. Additionally, therapeutic drug monitoring of tacrolimus levels was increased to twice weekly for the first month and weekly for the next 2 months, before resuming laboratory workup per transplant protocol (twice every month until 6 months after transplant, then monthly). He required transient reduction of his tacrolimus dose from 2 mg to 1 mg twice daily (bid) because of supratherapeutic trough levels (highest: 18 mg/dL, post-transplant goal: 7 mg/dL), one week after starting GLE/PIB. After 3 weeks, the tacrolimus dose was again increased to 2 mg bid (Figure 3). He achieved sustained virologic response (SVR) 12 weeks after treatment completion (SVR 12). He developed high-level BK viremia 1.5 year after transplant, which resolved by decreasing the tacrolimus trough goal to 4 mg/dL. Two years after transplant, he is doing well, and has excellent graft function and undetectable HIV, HCV, and HBV viral loads. He remains on entecavir prophylaxis.

## 3. Discussion

Management of patients with HIV and HCV/HBV tri-infection in the setting of ESRD presents unique challenges, including increased cardiovascular mortality, rapid progression of end organ dysfunction, infections, and DDI. Moreover, the rapidly changing landscape of HCV management in the DAA era has important implications for organ allocation. To our knowledge, this is the first report of an HIV/HCV/HBV coinfected patient with the non-1 HCV genotype who received an HCV-infected organ transplant with the discordant genotype. The lessons learnt are of clinical significance.

Cardiovascular disease and rates of myocardial infarction are already increased in patients with ESRD [29], but possibly further potentiated by HIV/HCV coinfection [30], by way of chronic inflammation, endothelial dysfunction, metabolic derangements [30–34], and potential choice of antiretroviral therapy [35]. Assessing cardiovascular risk in the pretransplant evaluation is imperative. Given the burden of cardiovascular disease in the ESRD population and frequent lack of symptoms, noninvasive cardiac imaging might be inferior to cardiac catheterization. In one study of kidney transplant candidates with cardiovascular risk factors other than HIV/HCV, the positive and negative predictive values of noninvasive means in detecting clinically significant cardiovascular disease were only 43% and 47%, respectively. Furthermore, mortality was reduced in those who underwent elective revascularization pretransplantation [29]. At our institution, following this case, we have a very low threshold for coronary angiography in HIV-infected or HCV-infected transplant candidates with a history of significant CAD, regardless of reported symptoms.

Management of DDI in patients coinfected with HIV and HCV is paramount, even more so in the setting of organ transplantation [3–5, 7, 13]. Fewer DDI are probably one of the main factors responsible for improved outcomes with InSTI compared to PI after organ transplantation in patients with HIV [4]. Coadministration of GLE/PIB with systemic tacrolimus (1 mg single dose) has been shown to increase tacrolimus *C*_max_ and area under the curve (AUC) by 1.5-fold and 1.45-fold, respectively. There was no change in the *C*_max_, AUC, and *C*_min_ of GLE or PIB [36]. As tacrolimus has a narrow therapeutic index, the official recommendation is to use with caution and with therapeutic drug monitoring, but without a priori dose adjustment. In general, improved liver function with anti-HCV treatment may further contribute to increased clearance of tacrolimus. In agreement with the above, our patient experienced an increase in tacrolimus trough level with initiation of GLE/PIB, resulting in dose reduction and subsequently decreased levels. Therefore, during coadministration of GLE/PIB and tacrolimus, we favor checking tacrolimus trough levels initially twice weekly and then weekly for the duration of treatment and a few weeks after, before resuming our regular per protocol laboratory workup.

In the setting of donor and recipient genotype strain mismatch, both viral strains may exist in the perioperative period. However, over the first few months post-transplantation, one strain will generally predominate although long-term detection of both genotypes is possible [19, 37, 38]. Recommended regimens for the treatment of HCV in kidney transplant recipients include ledipasvir/sofosbuvir, velpatasvir/sofosbuvir, and GLE/PIB. All are active against the most frequent genotype (1), and velpatasvir/sofosbuvir and GLE/PIB are pangenotypic and should be preferred in transplant recipients with donor/recipient genotype mismatch. It should be noted that strains with genotype 1 are likely more dominant (1b > 1a > 2), based on limited data, which is mostly derived from liver transplant recipients [37, 38]. This was confirmed in our case, despite kidney, not liver, transplantation, and therefore, substantially lower donor genotype 1a, compared to recipient genotype 2, viral load burden.

The optimal timing of DAA treatment in HCV-positive transplant candidates, including HIV coinfected patients, remains in a state of flux, with limited real-world data [7, 9, 10]. The risk of treatment delay and progression of liver disease, which can be accelerated by immunosuppression, should be weighed against the benefit of shorter wait time for HCV-infected organs. Based on sophisticated modelling, it has been suggested that for HCV-monoinfected patients with minimal liver fibrosis, treatment post-transplantation with an HCV-positive organ is beneficial. Nevertheless, in patients with advanced fibrosis, treatment prior to transplant may be preferred, unless the wait time benefit is >9 months [10]. For HCV/HIV coinfected patients, treatment post-transplant was consistently cost-saving as compared to treatment pretransplant and associated with higher life months and quality-adjusted life months with wait times >18 months [7]. At the time of our case, the wait time within our organ procurement organization (OPO) was <6 months vs. >4 years for HCV-positive vs. negative deceased donors, respectively. Thus, despite our patient's advanced stage of fibrosis, the decision to wait was justified.

In summary, our case study highlights the following: (1) transplant centers need to monitor OPO-specific times on the waitlist for an HCV-infected organ and to assess regularly the risk of delaying treatment for HCV-infected transplant candidates in anticipation of transplant from an HCV-infected donor; (2) closer monitoring of tacrolimus levels (once or twice weekly) during the early phases of anti-HCV PI introduction and discontinuation may be indicated; (3) the possibility of donor genotype transmission makes the use of pangenotypic DAA post-transplant appealing as the standard of care; (4) HIV/HCV coinfected transplant candidates require a holistic approach with emphasis on the cardiovascular risk profile and low threshold for cardiac catheterization as part of their pretransplant evaluation.
